# Benchmarking Probabilistic Time Series Forecasting Models on Neural Activity

**Published:** 2025-10-22

**Authors:** Ziyu Lu, Anna J. Li, Alexander E. Ladd, Pascha Matveev, Aditya Deole, Eric Shea-Brown, J. Nathan Kutz, Nicholas A. Steinmetz

**Affiliations:** 1 Department of Applied Mathematics, University of Washington, Seattle, WA, USA.; 2 Department of Neurobiology and Biophysics, University of Washington, Seattle, WA, USA.; 3 Allen Institute for Brain Science, Seattle, WA, USA.; 4 Department of Electrical and Computer Engineering, University of Washington, Seattle, WA, USA.

## Abstract

Neural activity forecasting is central to understanding neural systems and enabling closed-loop control. While deep learning has recently advanced the state-of-the-art in the time series forecasting literature, its application to neural activity forecasting remains limited. To bridge this gap, we systematically evaluated eight probabilistic deep learning models, including two foundation models, that have demonstrated strong performance on general forecasting benchmarks. We compared them against four classical statistical models and two baseline methods on spontaneous neural activity recorded from mouse cortex via widefield imaging. Across prediction horizons, several deep learning models consistently outperformed classical approaches, with the best model producing informative forecasts up to 1.5 seconds into the future. Our findings point toward future control applications and open new avenues for probing the intrinsic temporal structure of neural activity.

## 1 Introduction

Predicting the future state of a system is central to understanding its underlying dynamics. Beyond its value from the basic science perspective, time series forecasting is also critical to a wide range of real-world applications – including weather prediction[1], renewable energy planning[2], and financial market analysis[3] – guiding decisions ranging from everyday choices to high-stakes operations. Among the most challenging and rewarding domains for forecasting is the brain – one of the most complex and sophisticated systems known to science. Accurate forecasting of brain activity can not only offer insights into the underlying neural mechanisms but also enable transformative applications, including early intervention for neurological disorders[4], the development of therapeutic brain stimulation paradigms[5], and the advancement of brain–machine interfaces[6].

The field of time series forecasting has evolved dramatically over the past decade, driven by the rise of machine learning and deep neural networks[7]. Several studies have assembled datasets across various domains including energy, sales, climate, and healthcare, and systematically assessed the performance of both modern deep learning-based models and classical statistical methods on them[8, 9, 10, 11, 12, 13, 14]. However, with the exception of one recent study[15] which focuses on brain activity of zebrafish, neural time series have been largely absent from established forecasting benchmarks, and their markedly different characteristics from the included series make it unclear whether prior conclusions would apply to them. For instance, neural recordings are often sampled or binned at seconds to milliseconds resolutions, whereas most benchmark datasets are sampled at much coarser intervals (hourly, daily, or monthly). Furthermore, while oscillations are an important feature of brain activity[16], there is also a substantial amount of activity that does not exhibit persistent trends or periodicity/seasonality. In contrast, many real-world time series, such as climate records or energy consumption, are dominated by these patterns. These differences highlight the importance of systematically reassessing forecasting methods in the context of neural data.

Among neural recordings from different species, mouse data bring many important opportunities for time series forecasting. Foremost among the reasons is their relevance for developing and testing future neural control systems: recent advances in optogenetics[17, 18, 19] have enabled targeted manipulation of brain activity in mice, making accurate forecasts immediately applicable to the design of closed-loop control systems[20]. Moreover, modern recording technologies – such as Neuropixels probes[21, 22] for high-density, single-neuron resolution measurements and widefield calcium imaging[23] for simultaneously capturing activity across all dorsal cortical areas – allow large-scale monitoring at multiple spatial and temporal scales. Additional modalities, including behavior tracking[24, 25], cell-type labeling[26], and connectivity mapping[27], can further enhance forecasting performance and expand the range of scientific questions that forecasting can address. Finally, the abundance of publicly available mouse neural datasets[28, 29] provides a unique opportunity to develop large-scale foundation models[30] for neural activity forecasting in mice.

## 2 Related Work

Forecasting has long been an important approach in studying neural dynamics. Many earlier models, while not explicitly trained to optimize forecasting accuracy, are generative in nature and therefore can produce one-step-ahead or multi-step-ahead forecasts in an autoregressive fashion (e.g. [31, 32, 33]). As forecasting was only a secondary objective in these works, evaluations were generally restricted to a fixed prediction horizon, without systematic assessment across multiple horizons, and lack comparisons against forecasting baselines, such as the average of past activity or models capable of making direct-multi-step forecasts. Another major line of forecasting studies in neuroscience is motivated by brain-machine interface applications, where predicting neural activity is closely tied to predicting behavior. In this setting, models have been trained to solely predict behavior [34, 35, 36] or to jointly predict neural activity and behavior [37, 38]. However, since such experiments often involve highly structured tasks (e.g., a monkey reaching to target), the resulting neural dynamics tend to be stereotyped (e.g., with rotational structure[39]), making them easier to forecast compared to less structured scenarios such as spontaneous activity. Forecasting accuracy has also been combined with other performance measures for both training and evaluation, for example in [40, 41].

More recently, a growing number of models have begun explicitly adopting forecasting accuracy as their primary training objective. For example, [42] proposed a graph neural network based model for multi-channel neural activity forecasting; [43] used a low-rank linear autoregressive model to predict neural responses to holographic photostimulation; [44] developed a transformer-based model leveraging multi-modal inputs to autoregressively predict neural responses to visual stimuli; [45] introduced a diffusion-based model for joint forecasting of neural activity and behavior across sessions and subjects; [46] proposed a convolutional neural network based video model to directly forecast future frames of neural imaging videos; and [47] demonstrated forecasting of spontaneous neural activity across multiple sessions and subjects with a forecaster in the form of multilayer perceptron. While presented as neuroscience-specific applications, these models are fundamentally instances of general time series forecasting methods. Nevertheless, only a few ([46, 47]) have compared their performance against models from the broader time series forecasting literature, and even in those cases, the comparisons were limited. For example no recent foundation models (e.g., [48, 49]) were included. Moreover, all of these models produce only point forecasts. However, given the measurement and intrinsic noise in neural data [50], probabilistic forecasting [51] – which provides prediction intervals to quantify uncertainty – is particularly important, but remains unexplored in the context of neural time series forecasting.

To fill these gaps, we systematically benchmarked common baseline methods, classical statistical approaches, and state-of-the-art deep learning models from the probabilistic time series forecasting literature on spontaneous mice neural activity. By drawing on the broader forecasting literature, we aim to identify strong model backbones that future neuroscience-specific applications can build upon for more accurate and uncertainty-aware neural activity predictions.

## 3 Results

**Figure 1:**
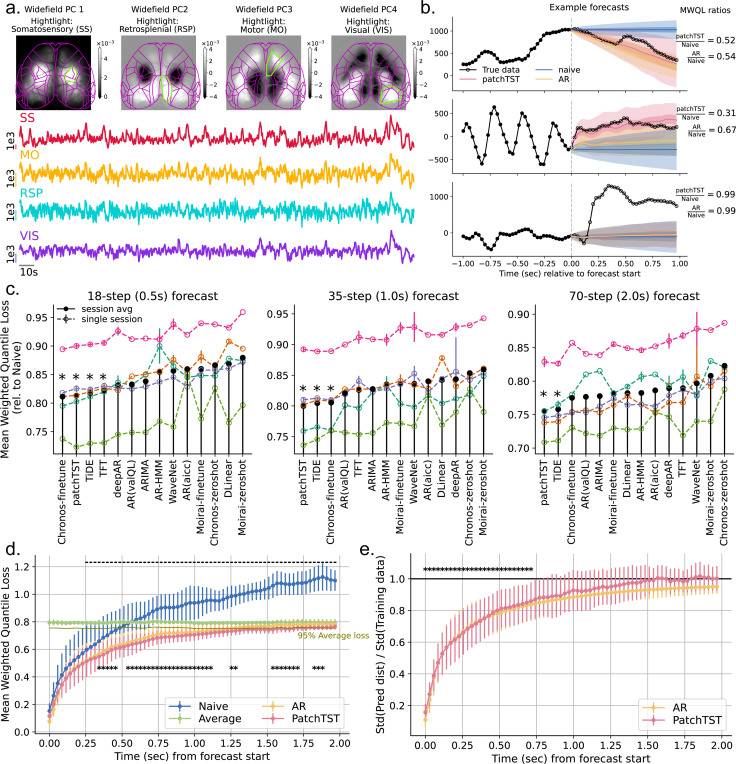
**a.** Widefield imaging data aligned to Allen CCF, with example activity traces from one session. **b.** True and predicted activity in three test samples; dashed lines mark forecast start. Shaded regions: prediction intervals (P.I.): dark = 20%, light = 60%. Top: PatchTST and AR perform similarly, both outperforming Naive. Middle: PatchTST outperforms both AR and Naive. Bottom: PatchTST and AR perform comparably to Naive. Quantitative performance in each sample is listed on the right. **c.** Model performance at three forecast horizons. Models sorted by mean performance across sessions. The y-axis indicates relative performance, computed as the Mean Weighted Quantile Loss (MWQL, see AppendixA.2 for definition) across all predicted steps of each model divided by that of the Naive baseline (i.e., y=1 corresponds to Naive performance). Colors indicate different sessions. Errorbar: mean ± std across 5 random seeds; fine-tuning Chronos yielded identical results across random seeds, likely due to an issue in the AutoGluon implementation. Performances of Theta and Average are omitted, as they are close to Naive and worse than all other models shown; for 70-step forecast, Average outperforms Naive but still not the others. AR(aicc) vs. AR(valQL): order chosen by AICC vs. validation MWQL. Models with stars significantly outperform AR(valQL) (one-sided paired t-test, *p* < 0.05). **d.** MWQL by forecast step. Errorbar: mean ± std across sessions. Bars and stars indicate steps where PatchTST significantly outperforms Naive and AR, respectively (one-sided paired t-test, *p* < 0.05). PatchTST and AR losses exceed 95% of the Average model loss (solid line) after 1.80s and 1.28s, respectively. **e.** Ratio of predicted distribution standard deviation (std) to training data std across forecast steps. Errorbar: mean ± std across sessions. Stars indicate steps where the PatchTST ratio is significantly less than 1 (one-sided t-test, *p* < 0.05). In **b**, **d**, **e**, AR(valQL) is used as AR. For PatchTST, in each session the model achieving median MWQL across 5 random seeds is used.

We systematically evaluated 12 univariate probabilistic forecasting models and 2 baseline methods on spontaneous neural activity in mouse cortex recorded using widefield calcium imaging[23] from five experimental sessions (details in AppendixA.1). Such data can be combined with cortex-wide optogenetic perturbations[18] to test forecast-driven closed-loop control. Widefield imaging was performed at 35 Hz, yielding recordings of spontaneous activity lasting 24.5 ± 3.7 minutes (corresponding to 51,495 ± 7,784 timesteps, mean ± std across five sessions). Data were registered to the Allen Mouse Brain Common Coordinate Framework (CCFv3)[52], and we extracted average activity traces from four major brain regions: somatosensory (SS), motor (MO), visual (VIS), and retrosplenial (RSP) (Figure 1a).

For baselines, we consider the Naive method which repeats the last observed activity, and the Average method which takes the mean of past observed activity as prediction. Among the 12 models, 4 are classical statistical methods: the autoregressive (AR) model, autoregressive integrated moving average (ARIMA)[53], autoregressive hidden Markov model (AR-HMM)[54], and the Theta model[55]; 6 are deep learning based models: DeepAR[56], DLinear[57], Temporal Fusion Transformer (TFT)[58], PatchTST[59], Time-series Dense Encoder (TiDE)[60], and WaveNet[61]; and 2 are time series foundation models[30]: Chronos[48] and Moirai[49], for which we evaluated both zero-shot and fine-tuned performance. More details on models and training are provided in AppendixA.2, A.4.

The forecasting task was defined as predicting activity in the interval [i,i+L) from the preceding observations in [i−H,i), where L is the forecast horizon and H is the history length. Following previous forecasting literature[56, 58, 62], we partitioned all time series chronologically, using the earliest 60% of timesteps for training $\left(\left[0,T_{\text{train}}\right)\right.$), the next 20% for validation $\left(\left[T_{\text{train}},T_{\text{val}}\right)\right.$), and the final 20% for testing $\left(\left[T_{\text{val}},T_{\text{test}}\right)\right.$). Validation and test samples were generated using sliding windows with non-overlapping targets. For instance, the first test sample forecasts $\left[T_{\text{test}},T_{\text{test}}+L\right)$ from $\left[T_{\text{test}}-H,T_{\text{test}}\right)$, the second forecasts $\left[T_{\text{test}}+L,T_{\text{test}}+2L\right)$ from $\left[T_{\text{test}}+L-H,T_{\text{test}}+L\right)$, and so on. In this way the forecast targets for evaluation remain fixed while varying the history length. We experimented with L=18, 35, and 70, which correspond to about 0.5, 1, and 2 seconds. H is treated as a hyperparameter and is tuned for each L and each model individually.

In aggregated performance over test samples, all methods outperformed the Naive and Average controls (Figure 1c, also see AppendixA.5, Figure 2 for evaluation on additional metrics). Classical autoregressive models provided competitive baselines, but several deep learning models – PatchTST, TiDE, and fine-tuned Chronos – consistently achieved higher accuracy across different prediction horizons. The strong performance of PatchTST aligns with findings from a recent benchmarking study on time series from various domains, such as economics, energy, and retail[14]. In contrast, foundation models (Chronos and Moirai) pretrained on these domains transferred poorly to neural activity in a zero-shot setting, indicating that neural time series exhibit distinct characteristics from the data used for pretraining. Nevertheless, Chronos becomes competitive after fine-tuning on neural activity, suggesting that its architecture is sufficiently flexible to capture neural dynamics. At the level of individual test samples, we found that while some features of activity were captured by the models, there also exist large fluctuations that were not predicted by any model (Figure 1b).

The improved performance of models over controls extended across a range of forecast steps, out to more than 1 second. Note that in Figure 1c, model performance is aggregated across all predicted steps. Therefore, it is actually unclear whether models still outperform baselines specifically at step 70, as the advantage may arise from better performance at earlier steps. When ploting error as a function of prediction step (Figure 1d), we observed that performance of Naive, AR, and PatchTST all deteriorates with increasing horizon. While the Naive model continues to worsen, AR and PatchTST converge toward the Average model’s score. This behavior is expected for AR models: it can be verified that for stationary time series, h-step-ahead AR forecast will converge to the mean as h→∞. A similar pattern can be found in other metrics (AppendixA.5, Figure 3). As PatchTST predicts all future steps simultaneously rather than autoregressively, separate models are optimized for each forecast horizon (18, 35, and 70 steps). We verified that the step-wise error curves are consistent across these models: for example, the first 18 steps of the 35- and 70-step models closely overlap with the 18-step model (AppendixA.5, Figure 4).

In addition, we quantified the uncertainty of probabilistic forecasts across steps, computed as the standard deviation of the predicted distribution over that of the training data (Figure 1e). For both AR and PatchTST, this ratio of standard deviations increases with forecast horizon and approaches one (which is again expected for the AR model). Importantly, while wider prediction intervals (as suggested by larger ratio here) may be less informative for encompassing too many possibilities, intervals that are narrower are not necessarily better, as they may miss sources of uncertainty[63]. Figure 1d, e suggest that forecasts are most reliable within 35 steps (1 sec), and informative predictions may extend to about 50 steps (1.5 sec); beyond this, even the best of the current models (PatchTST) performs no better than simply predicting the mean and standard deviation of training data.

## 4 Discussion

Several recent studies have proposed models tailored to forecasting neural time series. Nevertheless, models developed in the broader forecasting literature have not been systematically evaluated on neural activity, and the important aspect of uncertainty quantification has been largely overlooked. Here we bridged this gap by benchmarking models ranging from classical autoregressive methods to modern deep learning and foundation models on widefield imaging data. Several deep learning models consistently outperformed the strong baseline set by classical statistical models, yet both accuracy and uncertainty estimates indicate that current forecasts may only be informative up to a certain horizon. Whether this limit comes from model design constraints of existing methods or instead reflects intrinsic sources of variability and time scales in neural activity remains an open question. To probe the former, it may be of interest to develop neural time series forecasting foundation models by combining strong backbones identified here with neuroscience-specific innovations, such as cross-subject training[34, 36, 47, 45]. Meanwhile, closed-loop control experiments informed by forecasting performance may help reveal the temporal structure inherent to neural system.

## Supplementary Material

Supplement 1
