# Supplementary material for: Benchmarking Probabilistic Time Series Forecasting Models on Neural Activity
Source: ArXiv. 2025 Oct 22:arXiv:2510.18037v2. Preprint. [Version 2] (PMC12633634)
Supplement: Supplement 1 [file NIHPP2510.18037v2-supplement-1.pdf]

A Appendix

A.1 Data collection and preprocessing

All experimental protocols were conducted according to US National Institutes of Health guidelines for animal research and approved by the Institutional Animal Care and Use Committee at the University of Washington.

We analyzed five sessions of widefield imaging experiments collected from five male and female mice from multiple GCaMP-expressing genotypes: two expressed GCaMP8s (tetO-GCaMP8s), one expressed GCaMP6s (tetO-GCaMP6s), and two expressed jGCaMP7f (Ai210 and Ai210 triple). Some of the data has been used in [18] or [64], and the readers should refer to them for more experimental details. Briefly, images were collected with a CMOS camera with 17.3 um/pixel resolution (Basler acA2440-75m) and 560×560 pixel frame size. The camera was fitted with a 0.63x objective lens (Leica Planapo 0.63x). The true frame rate is 70 Hz, but the effective rate is 35 Hz due to alternating 405 nm and 470 nm excitation for hemodynamic correction. Mice were head-fixed facing a dark screen, and were free to turn a wheel. In two of the five sessions, 10% sucrose rewards were given in random 2-5 seconds intervals to keep mice at an awake and alert state. Three of the five sessions were solely spontaneous activity. In the other two sessions, receptive field mapping experiments were performed prior to spontaneous activity recording, and we analyzed spontaneous activity starting at least 2 minutes after the receptive field mapping experiments were turned off. Non-neuronal signals arising from hemodynamic changes were removed by subtracting the violet-light evoked signals from the blue-light evoked signals with linear regression, as previously described in [17, 64]. To store and process the data, we compressed the widefield data (D) into spatial components (U) and temporal components ($SV^\top$) with singular value decomposition in the form $D = USV^\top$. For each of the four brain regions (SS, MO, RSP, VIS), activity was reconstructed using the top 500 (four sessions) or 200 (one session) SVD components and was averaged across pixels within that region. It was reported in [18] and [64] that over 97% of the total variance before SVD compression was captured within the top 50 components. As we have used hundreds of components here, the information lost in compression and reconstruction should be negligible.

A.2 Univariate time series models

A.2.1 Baselines [65]

1. **Naive (repeat-last-step).** Let y_t denote the time series at timestep t, and $\{\epsilon_t\}$ be white noise with mean 0 and variance σ^2. The Naive baseline assumes y_t follows a random walk: $y_t = y_{t-1} + \epsilon_t$. Thus, to forecast from y_T, we have

$$\begin{aligned} y_{T+1} &= y_T + \epsilon_{T+1} \\ y_{T+2} &= y_{T+1} + \epsilon_{T+2} = y_T + \epsilon_{T+1} + \epsilon_{T+2} \\ &\cdots \\ y_{T+h} &= y_T + \sum_{i=1}^{h} \epsilon_{T+i} \end{aligned}$$

Let $y_{T+h|T}$ denote the forecast of y_{T+h} using previous observations up to (and including) y_T. It follows that

$$\begin{aligned} \hat{y}_{T+h|T} &:= \mathbb{E}[y_{T+h|T}] = y_T, \\ \hat{\sigma}_h^2 &:= \mathrm{Var}(y_{T+h|T}) = \mathrm{Var}(\sum_{i=1}^{h} \epsilon_{T+i}) = \sum_{i=1}^{h} \mathrm{Var}(\epsilon_{T+i}) = h\sigma^2, \end{aligned}$$

where σ^2 can be estimated from fitted residuals:

$$\sigma^2 = \frac{1}{T-1} \sum_{i=2}^{T} (y_i - y_{i|i-1})^2 = \frac{1}{T-1} \sum_{i=2}^{T} (y_i - y_{i-1})^2.$$

Assuming ϵ_t is normal, then a $100(1-\alpha)\%$ prediction interval is

$$\left[\hat{y}_{T+h|T} - z_{\frac{\alpha}{2}} \hat{\sigma}_h, \hat{y}_{T+h|T} + z_{\frac{\alpha}{2}} \hat{\sigma}_h\right],$$

where $z_{\frac{\alpha}{2}}$ is the value above which a fraction of $\frac{\alpha}{2}$ of the data in a standard normal distribution falls.

2. **Average.** Again let y_t denote the time series at timestep t, and $\{\epsilon_t\}$ be white noise with mean 0 and variance σ^2. The Average baseline assumes y_t randomly fluctuates around some mean value: $y_t = c + \epsilon_t$, where c is some constant to be estimated from the past observations. To forecast from y_T, we first compute the least-squares estimate of c:

$$\hat{c} = \frac{1}{T}\sum_{j=0}^{T-1} y_{T-j},$$

then for $i = 1, 2, \ldots,$

$$y_{T+i} = \hat{c} + \epsilon_{T+i}.$$

It follows that

$$\hat{y}_{T+h|T} := \mathbb{E}[y_{T+h|T}] = \hat{c},$$
$$\hat{\sigma}_h^2 := \mathrm{Var}(y_{T+h|T}) = \mathrm{Var}(\hat{c}) + \mathrm{Var}(\epsilon_{T+i}) = \frac{1}{T}\sigma^2 + \sigma^2,$$

where σ^2 can be estimated from fitted residuals:

$$\sigma^2 = \frac{1}{T-1}\sum_{j=0}^{T-1}(y_{T-j} - \hat{c})^2.$$

The prediction interval can be constructed in the same manner as in the Naive method. Note that $\hat{\sigma}_h$ is actually independent of h, and thus the width of prediction interval is constant through all forecasting steps.

A.2.2 Local models

The following models are "local" in the sense that we need to fit separate models to each series if there are multiple time series in one dataset.

1. **Autoregressive integrated moving average (ARIMA) family [53].** Let y_t denote the time series at timestep t, and $\{\epsilon_t\}$ be white noise. An ARIMA(p, q, d) model is of the form

$$y'_t = c + \phi_1 y'_{t-1} + \cdots + \phi_p y'_{t-p} + \theta_1 \epsilon_{t-1} + \cdots + \theta_q \epsilon_{t-q} + \epsilon_t$$

where y'_t is a d-th order differenced version of y_t. We applied both the KPSS test and the augmented Dickey–Fuller test to each time series here and found that they all satisfy the stationarity condition. So we take $d = 0$, $y'_t = y_t$, and the ARIMA model simplifies to an ARMA model. The **autoregressive (AR) model** can be seen as a special case of ARIMA, where $d = 0$, and the $\theta's$ are taken to be zero. During fitting, for fixed p, q, the parameters c, ϕ_i, θ_i are determined by maximizing the likelihood of observed data under the assumption that $\{\epsilon_t\}$ is Gaussian

$$L(c, \{\phi_i\}_{i=1}^p, \{\theta_i\}_{i=1}^q) = \prod_{t=1}^n f(y'_t | y'_{t-1}, \ldots, y'_1, c, \{\phi_i\}_{i=1}^p, \{\theta_i\}_{i=1}^q).$$

To find the most appropriate value of (p, q), a common approach is to compute the likelihood for various values of (p, q), and choose the pair achieving the minimum AICC[66]. Alternatively, we can evaluate fitted models on a validation set, and selected the (p, q) pair achieving the best score on the validation set.
The **autoregressive hidden Markov model (AR-HMM)** extends the AR model, allowing the parameters to switch among different values. It can be written as

$$y_t = c^{S_t} + \phi_1^{S_t} y_{t-1} + \cdots + \phi_p^{S_t} y_{t-p} + \epsilon_t^{S_t},$$

where S_t denotes the state (assumed to be hidden, unobserved) at timestep t, and $\epsilon_t^{S_t} \sim \mathcal{N}(0, (\sigma^{S_t})^2)$. S_t switches among a set of discrete values following a first-order Markov chain. Since it is possible to transition into several different states at each step (although some states are more probable than others), AR-HMM can make probabilistic forecasts by sampling multiple trajectories from the forecast start.

2. **Theta family [55, 67].** The general idea of Theta model is to decompose the time series into multiple components with different curvatures (as controlled by second derivatives), extrapolate each component separately into the future, and then combine their extrapolations. In its most basic form proposed in [55], two Theta lines are drawn from the original time series: one with second derivative equal to 0 ($\Theta = 0$) for extracting long-term linear trend, and the other with second derivative double that of the original series ($\Theta = 2$) for extracting local fluctuations. During prediction, the first Theta line ($\Theta = 0$) continues to follow the linear trend, and the second Theta line ($\Theta = 2$) is extrapolated via simple exponential smoothing. A simple average of their extrapolations is taken to be the final forecast. Methods to further improve Θ-parameter selection and extrapolations were later proposed in [67]. In a recent benchmark study [14], the Theta family demonstrates competitive performance on time series with high sampling frequency (10 sec). Nonetheless, as those series are from a very different domain (cloud operations) than neuroscience, it is unclear whether the Theta family is also effective for forecasting widefield imaging data.

A.2.3 Global models

The following models are “global" in the sense that one model can simultaneous fit all time series in a dataset.

1. **DeepAR[56].** DeepAR model makes probabilistic forecasts with an autoregressive recurrent neural network (RNN). The value of the time series at the next timestep is assumed to be drawn from some probability distribution, which is usually taken to be Gaussian or Student-t for continuous data. The parameters of the distribution (e.g., the mean and variance for Gaussian distribution) are determined by the output of a multi-layer RNN: Let y_t denote time series observations and x_t denote some known covariate. At timestep t, the RNN computes $\boldsymbol{h}_t$ from $\boldsymbol{h}_{t-1}, y_{t-1}, x_t$, and assumes $y_t \sim \theta(\boldsymbol{h}_t)$, where $\theta(\cdot)$ is some fixed distribution. In practice, it is often advantageous to also incorporate lagged versions of y_{t-1} and x_t (e.g. y_{t-2}, x_{t-1}) when computing $\boldsymbol{h}_t$. During inference, to forecast L steps from $t = T + 1$, the model will first run from $t = T - H$ (where H is the relevant history context length) to T to generate $\boldsymbol{h}_{T+1}$, sample one $\hat{y}_{T+1}$ from $\theta(\boldsymbol{h}_{T+1})$, and then use $\hat{y}_{T+1}, \boldsymbol{h}_{T+1}, x_{T+1}$ to compute $\boldsymbol{h}_{T+2}$ and generate a sample trajectory in this autoregressive form. To make probabilistic forecasts, multiple sample trajectories will be generated, and the prediction interval at each time step will be estimated from the sample trajectories.
2. **DLinear[57].** DLinear model is a one-layer linear feedforward network for direct multi-step forecasting. It was originally proposed to showcase that the advantage of many transformer-based models in time series forecasting mainly comes from the direct multi-step-ahead forecasting strategy (as opposed to autoregressive one-step-ahead), instead of the transformer architecture. Suppose we want to forecast $\boldsymbol{y}^{\text{future}} = [y_T, \ldots, y_{T+L}]$ from $\boldsymbol{y}^{\text{history}} = [y_{T-H}, \ldots, y_T]$. Using a moving average smoother, $\boldsymbol{y}^{\text{history}}$ is first decomposed into a trend and a remainder component: $\boldsymbol{y}^{\text{history}} = \boldsymbol{y}^{\text{history, trend}} + \boldsymbol{y}^{\text{history, remainder}}$. Each of the two components is passed through a one-layer linear feedforward network, and then summed together as the future forecast: $\hat{\boldsymbol{y}}^{\text{future}} = \boldsymbol{W}_1 \boldsymbol{y}^{\text{history, trend}} + \boldsymbol{W}_2 \boldsymbol{y}^{\text{history, remainder}}$, where $\boldsymbol{W}_1, \boldsymbol{W}_2$ are $L \times H$ matrices. If the time series is multivariate, i.e., for the i-th variate, $\boldsymbol{y}^{(i),\text{future}} = \left[y_T^{(i)}, \ldots, y_{T+L}^{(i)}\right]$, $\boldsymbol{y}^{(i),\text{history}} = \left[y_{T-H}^{(i)}, \ldots, y_T^{(i)}\right]$, then $\boldsymbol{W}_1, \boldsymbol{W}_2$ will be shared across all variates and no cross-variate relationship will be modeled, i.e., $\hat{\boldsymbol{y}}^{(i),\text{future}} = \boldsymbol{W}_1 \boldsymbol{y}^{(i),\text{history, trend}} + \boldsymbol{W}_2 \boldsymbol{y}^{(i),\text{history, remainder}}$ for all i.

   The original DLinear model only produces point forecasts, but the GluonTS package[68] adds an additional simple transformation step to make it output the parameters of the desired distribution and thus make the forecasts probabilistic. For example, for the Gaussian distribution, $\hat{y}_t^{\text{future}} \sim \mathcal{N}(\mu(\boldsymbol{y}^{\text{feature}}), \sigma(\boldsymbol{y}^{\text{feature}}))$, where $\boldsymbol{y}^{\text{feature}} \in \mathbb{R}^D$. $\boldsymbol{y}^{\text{feature}} = \boldsymbol{W}_1 \boldsymbol{y}^{\text{history, trend}} + \boldsymbol{W}_2 \boldsymbol{y}^{\text{history, remainder}}$ as in the original DLinear model. $\mu(\boldsymbol{y}^{\text{feature}}) = \boldsymbol{w}_\mu^\top \boldsymbol{y}^{\text{feature}} + \boldsymbol{b}_\mu$, and $\sigma(\boldsymbol{y}^{\text{feature}}) = \log(1 + \exp(\boldsymbol{w}_\sigma^\top \boldsymbol{y}^{\text{feature}} + \boldsymbol{b}_\sigma))$.
3. **TFT[58].** Temporal fusion transformer (TFT) is a versatile model that can incorporate static covariates, covariates that are only known in the past, and covariates that are known for the forecasting horizon. It first uses recurrent layers to extract local temporal information that is shared across neighboring time steps, and then uses the attention mechanism to process global temporal information that are spread across a wider time frame. It performs

probabilistic forecasting by optimizing the quantile loss. However, one caveat is that it does not address the problem of quantile crossing[69]. For instance, the predicted 0.5-quantile may be larger than the predicted 0.9-quantile.

4. **PatchTST[59].** PatchTST model uses the vanilla transformer encoder[70] as backbone, together with two key designs: channel-independence and patching. Suppose we want to forecast a M-dimensional multivariate time series $\boldsymbol{y}^{\text{future}} = [\boldsymbol{y}_T, \dots, \boldsymbol{y}_{T+L}]$ from $\boldsymbol{y}^{\text{history}} = [\boldsymbol{y}_{T-H}, \dots, \boldsymbol{y}_T]$, where $\boldsymbol{y}_t \in \mathbb{R}^M$. PatchTST models each variate individually using a shared model $\mathcal{F}$ ("channel-independence"): $\boldsymbol{y}^{(i),\text{future}} = \mathcal{F}(\boldsymbol{y}^{(i),\text{history}})$, where $\boldsymbol{y}^{(i),\text{future}} = \left[y_T^{(i)}, \dots, y_{T+L}^{(i)}\right]$, $\boldsymbol{y}^{(i),\text{history}} = \left[y_{T-H}^{(i)}, \dots, y_T^{(i)}\right]$. For each variate i, neighboring timesteps are grouped together as one "patch" (or token), and then fed into the transformer encoder. The patches may or may not be overlapping, and the number of patches is typically smaller than the number of history context timesteps. Each patch will be linearly mapped to a high-dimensional vector, and go through nonlinear transformations (such as self-attention and feed-forward networks) in the transformer encoder. $\hat{\boldsymbol{y}}^{(i),\text{future}}$ is computed from linear transformation of the transformer encoder output. Similar to DLinear, the original PatchTST model only produces point forecasts, but is adapted for probabilistic forecasting by the GluonTS[68] package.

5. **TiDE[60].** Time-series Dense Encoder (TiDE) can be thought as a nonlinear extension of the DLinear model. While DLinear only accounts for the linear relationship, TiDE uses the classical multi-layer perceptron and residual connections to capture the potentially nonlinear relationship between future and observed activity. When there are multiple time series in the dataset, it operates in a channel-independent manner, as PatchTST, and does not model the relationship between different time series. Similar to DLinear and PatchTST, the original TiDE model only produces point forecasts, but is adapted for probabilistic forecasting by the GluonTS[68] package.

6. **WaveNet[61].** WaveNet is a convolutional neural network-based autoregressive model. For a sequence $[y_1, \dots, y_T]$, it learns $p(y_t|y_{t-1}, \dots, y_1)$ for all t. WaveNet is originally proposed for modeling audio signals, which are usually stored as 16-bit integers so only take a finite number of values. Thus WaveNet can learn $p(y_t|\cdots)$ as categorical distributions and can be trained using the cross-entropy loss. GluonTS[68] adapts WaveNet to model general time series by discretizing the real-valued observations into a fixed number of bins so they can be modeled as categorical distributions too. Similar to DeepAR, WaveNet generates probabilistic forecasts by autoregressively sampling different trajectories. In a recent study[71], WaveNet was applied to forecast resting-state EEG signal and was observed to outperform the classical AR model.

A.2.4 Foundation models

1. **Chronos[48].** Chronos is a large language model (LLM)-based method for forecasting univariate time series, using the T5 model[72] as backbone. The idea is that, upon proper tokenization, time series can be forecast in the same way as text using LLM. Since LLMs work with a finite dictionary of tokens (i.e., words), Chronos discretizes a real-valued time series into a fixed number of bins, and is trained using the cross-entropy loss (similar to the adapted WaveNet). During prediction, the original Chronos model proposed in [48] follows the autoregressive one-step-ahead forecasting strategy, similar to DeepAR. The later version, Chronos-Bolt[73], adopts the direct multi-step-ahead strategy, which generates estimates of quantiles $0.1, \dots, 0.9$ of all predicted steps simultaneously using a feedforward network applied to decoder output. However, similar to TFT, Chronos-Bolt also faces the problem of quantile crossing[69].

   As with other LLMs, Chronos has been trained on a huge amount of data. Its training set consists of 28 real-world datasets with about 890K univariate time series spanning multiple domains (weather, finance, transportation, etc). It is trained with maximum context length of 512 and maximum forecast horizon of 64 time steps. Because of its rich training history, Chronos may be capable to perform zero-shot forecasting on time series that have never been seen by the model before. Nonetheless, since the majority of the time series in the training set of Chronos is of low frequency (sampled at hourly or lower rate), it is unclear whether the pretraining of Chronos can actually benefit it in forecasting widefield data.

2. **Moirai[49].** Moirai is another LLM-based time series forecasting model. Its main differences from Chronos are that it can handle time series with covariates and does not require discretizing time series into categorical variables. To achieve the former, it flattens the multivariate time series (e.g., a matrix of shape $d \times T$) into one sequence (e.g. a vector of length $d \times T$), while keeping track of the variate identities and time steps. Also, similar to PatchTST, instead of defining each time step as a separate token input to the underlying transformer model, Moirai forms "patches" by grouping nearby time steps of the same variate, and take each patch as a token. The patch size is set based on the frequency of the data. To model time series with diverse distributions of values without mapping them to categorical variables, Moirai is trained by maximizing the log-likelihood with respect to a mixture of probability distributions:

$$l(\boldsymbol{\theta}) = \log p(y_{T+1}, \ldots, y_{T+h} | f_{\boldsymbol{\theta}}(y_{T-C}, \ldots, y_T)), \text{with } p(\cdot) = \sum_{i=1}^{4} w_i p_i(\cdot),$$

where the $w_i's$ are also learnable parameters and the $p_i's$ are taken to be the Student's t, negative binomial, log-normal, and normal distributions. Probabilistic forecasts can be generated by sampling from this mixed distribution. Just like Chronos, Moirai has also been trained on a huge amount of data with a total of 27 billion observations, and thus may be capable of zero-shot forecasting. During training, Moirai uses samples with randomized history and prediction length to further improve its applicability to diverse forecasting problems.

A.3 Metrics

Let $y_{i,t}$ denote the true activity at t-th forecast step in the i-th test sample, $y_{i,t} \sim D_{i,t}$, $t = 1, \ldots, H, i = 1, \ldots, N$. Let $f_{i,t}^q$ denote the predicted q-th quantile of $D_{i,t}$.

1. **Mean Weighted Quantile Loss (MWQL):** When aggregating across all test samples and prediction steps (Figure 1c), we computed

$$\text{MWQL} = \frac{\sum_{i=1}^{N} \sum_{t=1}^{H} \frac{1}{Q} \sum_q \rho_q(y_{i,t}, f_{i,t}^q)}{\sum_{i=1}^{N} \sum_{t=1}^{H} |y_{i,t}|}.$$

When aggregating across all test samples for each prediction steps t (Figure 1d), we computed

$$\text{MWQL}_t = \frac{\sum_{i=1}^{N} \frac{1}{Q} \sum_q \rho_q(y_{i,t}, f_{i,t}^q)}{\sum_{i=1}^{N} |y_{i,t}|}.$$

In both cases, $q = 0.1, 0.2, \ldots, 0.9$, $Q = 9$. ρ_q is the quantile score[62]:

$$\rho_q(y_{i,t}, f_{i,t}^q) = \begin{cases} 2(1-q)(f_{i,t}^q - y_{i,t}), & \text{if } y_{i,t} < f_{i,t}^q \\ 2q(y_{i,t} - f_{i,t}^q), & \text{if } y_{i,t} \geq f_{i,t}^q \end{cases}$$

MWQL has been popularly used for evaluating probabilistic forecasts, e.g., in [69, 48, 49].

2. **Mean Scaled Interval Score (MSIS):**

$$\text{MSIS} = \frac{1}{N} \sum_{i=1}^{N} \frac{\sum_{t=1}^{H} W_{i,t}^{\alpha}(y_{i,t}, u_{i,t}^{\alpha}, l_{i,t}^{\alpha})}{\sum_{t=1}^{H} |y_{i,t}|}$$

Here $[l_{i,t}^{\alpha}, u_{i,t}^{\alpha}]$ define a $100(1-\alpha)\%$ prediction interval for $y_{i,t}$, typically $l_{i,t}^{\alpha} = f_{i,t}^{\frac{\alpha}{2}}$, $u_{i,t}^{\alpha} = f_{i,t}^{1-\frac{\alpha}{2}}$. W^{α} is the Winkler score[62], which combines penalties for the width of the interval and for lack of coverage:

$$W^{\alpha}(y_{i,t}, u_{i,t}^{\alpha}, l_{i,t}^{\alpha}) = (u_{i,t}^{\alpha} - l_{i,t}^{\alpha}) + \frac{2}{\alpha}(l_{i,t}^{\alpha} - y_{i,t})\mathbb{1}(y_{i,t} < l_{i,t}^{\alpha}) + \frac{2}{\alpha}(y_{i,t} - u_{i,t}^{\alpha})\mathbb{1}(y_{i,t} > u_{i,t}^{\alpha})$$

In this paper we took $\alpha = 0.2$. [69, 49] used a similar metric to evaluate prediction intervals, where the denominator is the absolute seasonal error. Since seasonality is not obvious in neural time series, we used the absolute target value instead.

3. **Mean Absolute Error (MAE) and Mean Squared Error (MSE):** When aggregating across all test samples and prediction steps (Figure 2), we computed

$$\text{MAE} = \frac{\sum_{i=1}^{N}\sum_{t=1}^{H}|y_{i,t}-\hat{y}_{i,t}|}{\sum_{i=1}^{N}\sum_{t=1}^{H}|y_{i,t}|},\ \text{MSE} = \frac{\sum_{i=1}^{N}\sum_{t=1}^{H}|y_{i,t}-\hat{y}_{i,t}|^2}{\sum_{i=1}^{N}\sum_{t=1}^{H}|y_{i,t}|^2}$$

   When aggregating across all test samples for each prediction steps t (Figures 3,4), we computed

$$\text{MAE}_t = \frac{\sum_{i=1}^{N}|y_{i,t}-\hat{y}_{i,t}|}{\sum_{i=1}^{N}|y_{i,t}|},\ \text{MSE}_t = \frac{\sum_{i=1}^{N}|y_{i,t}-\hat{y}_{i,t}|^2}{\sum_{i=1}^{N}|y_{i,t}|^2}$$

   Here $\hat{y}_{i,t}$ is a point forecast of $y_{i,t}$, and is taken to be the median value of probabilistic forecasts, i.e., $f_{i,t}^{0.5}$. MAE was also used in [56], where it was called Normalized Deviation.

4. **Correlation:** We computed the Pearson's correlation coefficient r between $y_i = \{y_{i,t}\}_{t=1,\dots,H}$ and $\hat{y}_i = \{\hat{y}_{i,t}\}_{t=1,\dots,H}$ for each test sample. The median r across of all test samples is reported is Figure 2.

A.4 Model implementation and training details

For the **Naive** and **Average** baselines, we used the implementations from the StatsForecast package [74]. These models have no trainable parameters or hyperparameters.

For the **AR** model, we considered two order–selection strategies:

1. AICC-based (**AR(aicc)**): we used `AutoARIMA` from StatsForecast, which by default uses the Hyndman-Khandakar stepwise search algorithm to find the model order and parameter achieving the lowest AICC[62]. Models were fitted using both training and validation sets. We set the limit on the number of models to explore to 100.
2. Validation-based (**AR(valQL)**): we used `AutoRegressive` from StatsForecast. Starting from lag order 1, we fitted the model on the training set and computed its MWQL on the validation set. The lag order was increased by one until validation MWQL failed to improve for 10 consecutive orders; the best-performing model so far was then chosen and evaluated on the test set.

For **ARIMA**, we again used `AutoARIMA` from StatsForecast. The AR order (p) was initialized with the optimal order found by **AR(aicc)**, and the MA order (q) was initialized to 0. Models were fitted using both training and validation sets. We set the limit on the number of models to explore to 100.

For **Theta**, we fitted four variants provided in StatsForecast (standard, optimized, dynamic standard, and dynamic optimized) on the training set. The variant with the lowest validation MWQL was evaluated on the test set.

For **AR-HMM**, we used `LinearAutoregressiveHMM` from the Dynamax package[75]. We fitted models with number of states $S \in \{2,3,4,5,6,7,8,9,10\}$ and number of lags $L \in \{1,2,4,6,\dots,40,44,48,\dots,80\}$ on the training set, and evaluated their log likelihoods of the validation data (LL_{val}). The optimal S was chosen as the smallest value whose LL_{val} was within 0.1% of the maximum across all models (i.e., $\geq 1.001 \times \max \text{LL}_{\text{val}}$, since LLs are negative). The optimal L was chosen as the smallest value whose LL_{val} was within 0.1% of the maximum across all lags at the selected S. We then fitted models with the optimal (S, L) with 5 random seeds on the training set and evaluated them on the test set. For each test sample, we generated 100 forecast trajectories to compute prediction intervals.

For **Global** models, we used the implementation from GluonTS[68]. Some models (**DLinear**, **PatchTST**, **TiDE**) was originally proposed for point forecast, but were adapted to generate probabilistic forecasts by GluonTS. See the **DLinear** section in A.2 for an example of such adaption. All models assumed Student-t output distributions. During training, all models employed early stopping based on validation loss, terminating if no improvement occurred for 10 epochs. Since widefield data does not clear seasonality, we disabled automatically added time features (e.g., hour of day, day of week) in **TiDE** and **WaveNet**. Hyperparameters were tuned by random search[76]: for each model, 40 hyperparameter configurations were randomly sampled from a grid, and the optimal configuration was selected as the one with the lowest validation MWQL. Finally, we trained models

with the optimal hyperparameter configuration using 5 random seeds, and evaluated them on the test set. Table 1 lists the names and candidate values of the hyperparameters for each model. Note that the values of `context_length` include all candidate values for all prediction lengths, but the candidates may vary across prediction lengths: for each session, we first performed hyperparameter search for forecasting 35 steps. Depending on the validation results, we may adjust the candidates for forecasting 70 and 18 steps. The candidate values of all other hyperparameters were the same across all sessions and prediction lengths.

For **Chronos**, we used `chronos-bolt-base` from AutoGluon[77]. For fine-tuning, we fixed `fine_tune_batch_size` $= 128$, `fine_tune_lr` $= 10^{-5}$, and varied `fine_tune_steps` $\in \{100, 200, 400, 600, 800, 1000\}$. The configuration achieving the lowest validation MWQL was then evaluated on the test set. Fine-tuning Chronos yielded identical results across random seeds, likely due to an issue in the AutoGluon implementation.

For **Moirai**, we used `moirai-1.0-R-base` from the official implementation of [49]. Since **Moirai** randomly samples prediction and context length during training, for zero-shot evaluation, we performed inference tuning as in [49]. Specifically, we varied context length $C \in \{50, 100, 250, 500, 750, 1000, 2000, 3000, 4000, 5000\}$ and patch size $P \in \{8, 16, 32, 64, 128\}$, and evaluated all pairs with the desired prediction horizon on the validation set. The pair with the lowest validation MWQL was then evaluated on the test set. Note that there is not a default patch size for the sampling frequency of widefield data, so we swept through all possible patch sizes of **Moirai**. 32 and 64 generally work the best. For fine-tuning, we fixed `batch_size` $= 64$ (due to memory constraint) and varied `learning_rate` $\in \{10^{-3}, 5 \times 10^{-4}, 10^{-4}, 5 \times 10^{-5}, 10^{-5}, 5 \times 10^{-6}, 10^{-6}\}$, while keeping other setting as default. The optimal learning rate was chosen as the one with the lowest validation loss when evaluated with the specific prediction length, `patch_sizes` $= [32, 64]$, and `context_lengths` $= [1000, 2000, 3000, 4000, 5000]$. The best hyperparameter configuration was then fine-tuned with 5 different random seeds. For each seed, we computed the validation MWQL across multiple (C, P) pairs as in the zero-shot setting. The pair with the lowest validation MWQL across 5 seeds was evaluated on the test set.

A.5 Supplementary figures

Table 1: Hyperparameter search space for GluonTS models.

Model	Hyperparameter	Values
**DeepAR**	lr	{2.5e-05, 5.0e-05, 7.5e-05, 1.0e-04, 7.5e-04}
	batch_size	{32, 64, 128}
	hidden_size	{64, 128, 256}
	num_layers	{1, 2, 3}
	context_length	{35, 70, 140, 175, 210, 245}
	lags_seq	{1,2, ..., L-1}, where $L \in \{5, 10, 20, 30, 40\}$
**PatchTST**	context_length	{35, 70, 140, 175, 210, 245}
	patch_len	{24, 32, 40}
	stride	$s \times$ patch_len, where $s \in \{0.25, 0.5, 1\}$
	d_model	{32, 64, 128, 256}
	nhead	{1, 4}
	dim_feedforward	$r \times$ d_model, where $r \in \{1, 2\}$
	activation	'relu'
	num_encoder_layers	{1, 2, 3}
	lr	{0.0001, 0.00025, 0.0005, 0.00075, 0.001}
	batch_size	{128, 256, 512}
**DLinear**	context_length	{140, 175, 210, 245, 280, 315}
	hidden_dimension	{16, 32, 64, 128, 256}
	lr	{0.0001, 0.0005}
	kernel_size	{5}
	batch_size	{128, 256, 512}
**TiDE**	context_length	{35, 70, 105, 140, 175, 210}
	feat_proj_hidden_dim	4
	encoder_hidden_dim	{64, 128, 256, 512, 1024}
	decoder_hidden_dim	same as encoder_hidden_dim
	temporal_hidden_dim	{64, 128}
	distr_hidden_dim	{4, 8, 16, 32, 64, 128}
	num_layers_encoder	{1, 2}
	num_layers_decoder	{1, 2, 3}
	decoder_output_dim	{4, 8, 16, 32}
	dropout_rate	{0.3, 0.5}
	num_feat_dynamic_proj	2
	layer_norm	False
	lr	{1.e-05, 5.e-05, 1.e-04, 5.e-04}
	batch_size	{128, 256, 512}
**TFT**	context_length	{70, 105, 140, 175, 210, 245, 280, 315}
	quantiles	[0.05, 0.1, 0.2, 0.3, 0.4, 0.5, 0.6, 0.7, 0.8, 0.9, 0.95]
	num_heads	{1, 2, 4}
	hidden_dim	{64, 128, 256}
	variable_dim	same as hidden_dim
	dropout_rate	{0.2, 0.4, 0.6}
	lr	{0.00075, 0.001, 0.0025}
	batch_size	{128, 256, 512}
**WaveNet**	num_bins	{1024, 2048}
	num_residual_channels	{4, 8, 12, 24, 36, 48}
	num_skip_channels	{4, 8, 16, 32, 48, 64}
	dilation_depth	{2, 4, 8, 10}
	num_stacks	{1, 2, 3}
	lr	{1.e-05, 5.e-05, 1.e-04, 5.e-04, 1.e-03}
	batch_size	{32, 64, 128, 256}

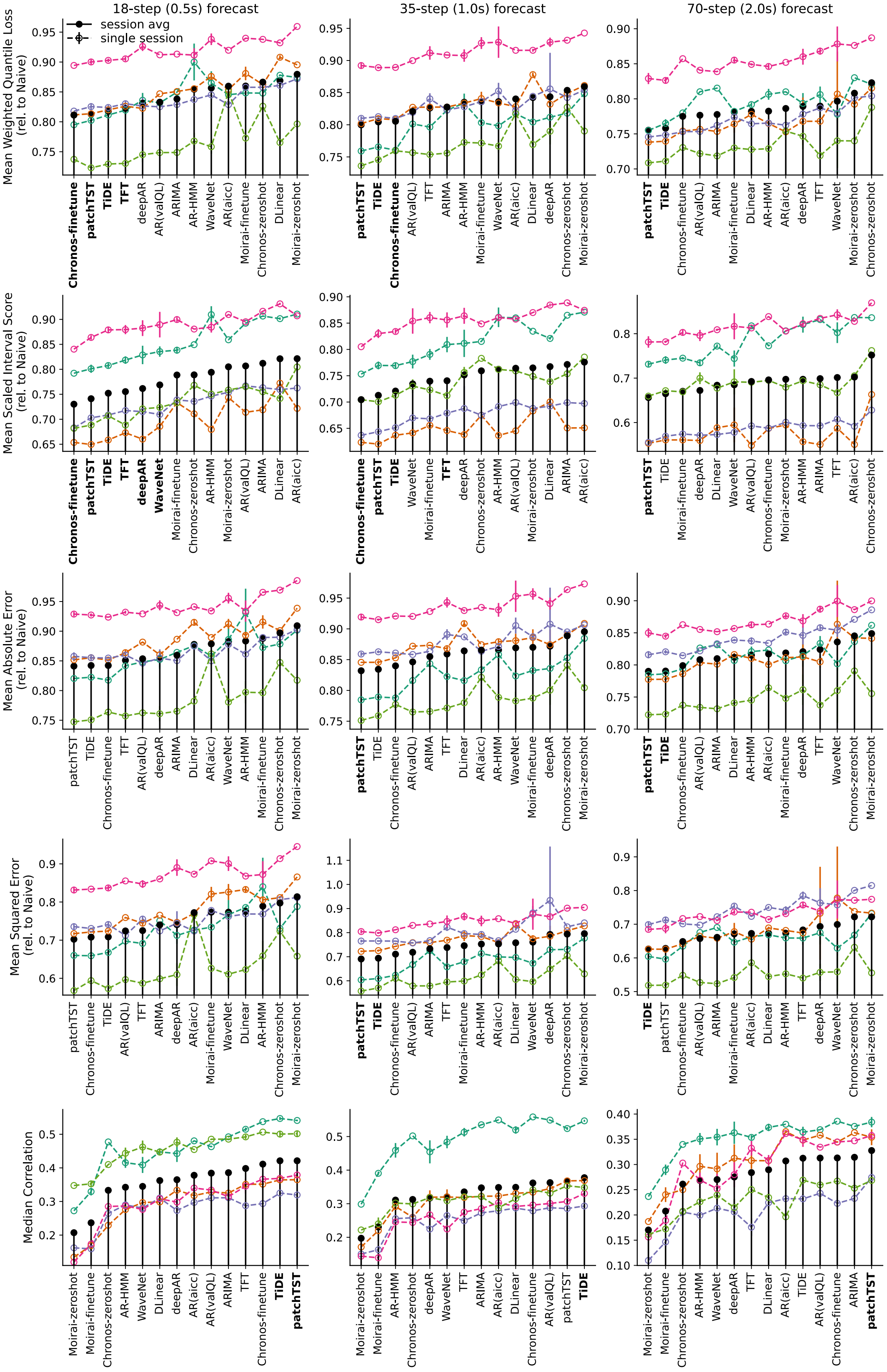

Figure 2: Evaluation on additional metrics. Models sorted by mean performance across sessions. Models in boldface significantly outperform AR(valQL) (one-sided paired t-test, $p < 0.05$). For Chronos and Moirai, -FT indicates the finetuned version, -ZS indicates zeroshot version.

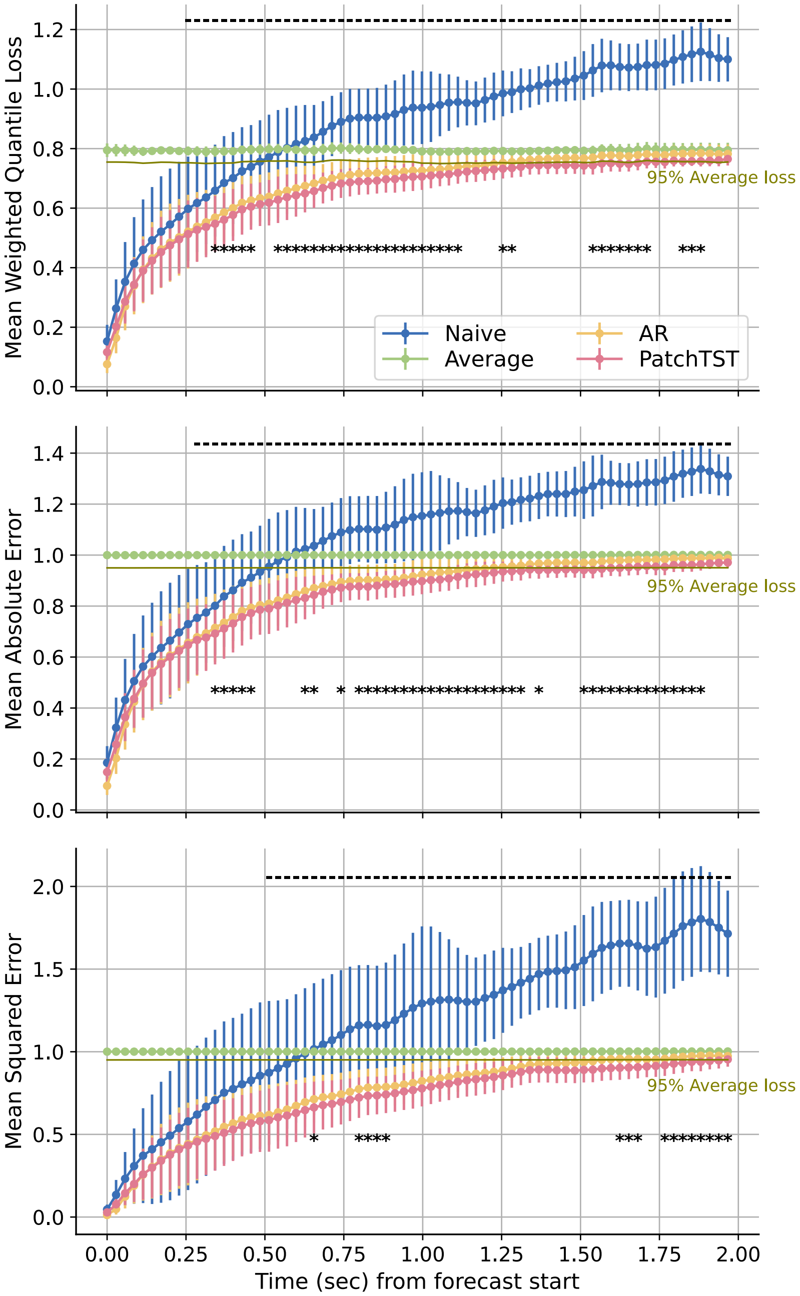

Figure 3: Additional metrics over prediction steps. Bars and stars indicate steps where PatchTST significantly outperforms Naive and AR, respectively (one-sided t-test, $p < 0.05$). PatchTST loss exceeds 95% of the Average model loss (solid line) after 1.80s (top), 1.65s (middle), 1.97s (bottom). AR loss exceeds 95% of the Average model loss after 1.28s (top), 1.23s (middle), 1.60s (bottom).

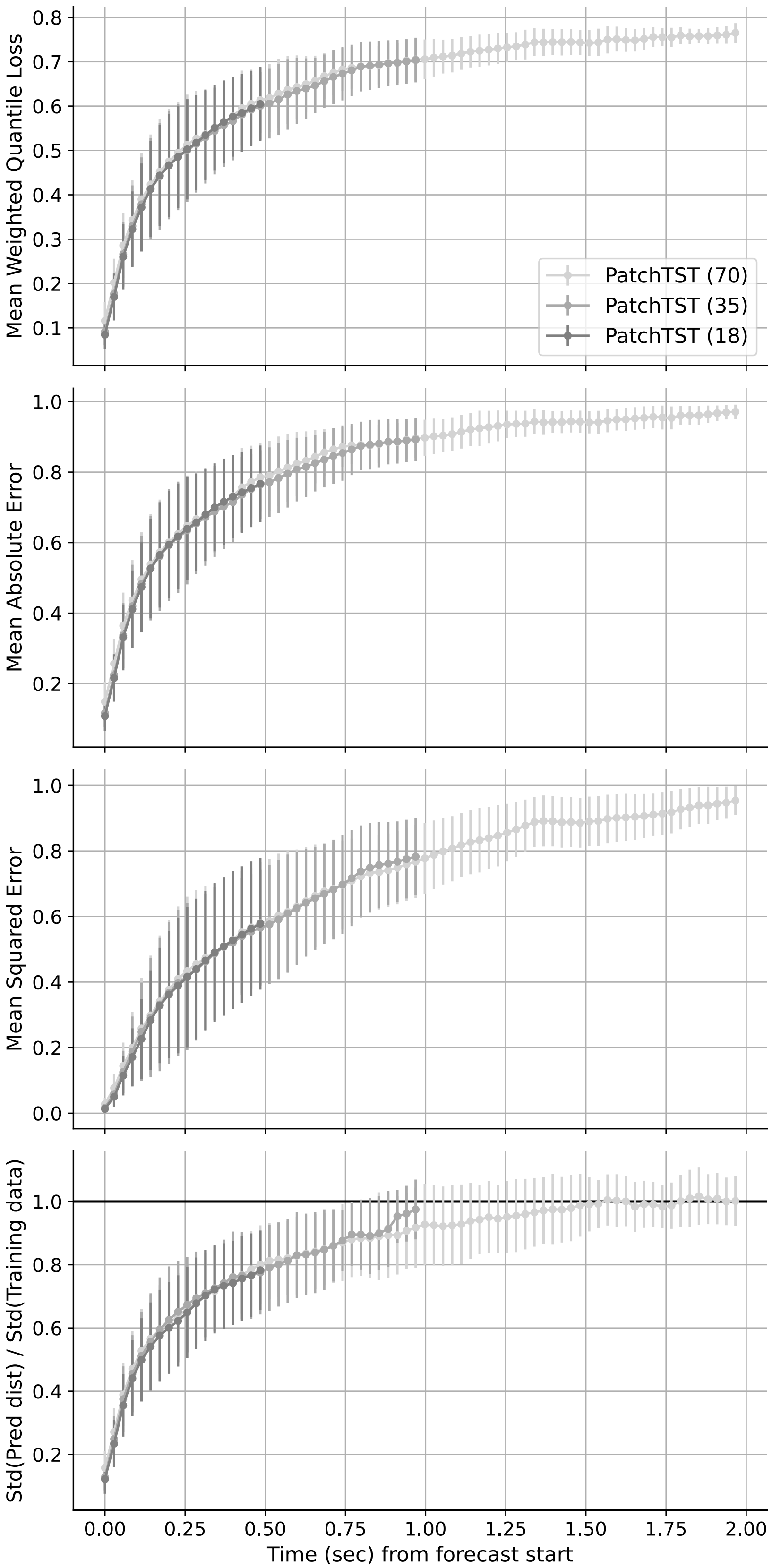

Figure 4: Performance across PatchTST models trained with different forecast horizons. PatchTST (18), PatchTST (35), PatchTST (70): PatchTST trained with forecast horizon of 18, 35, and 70 steps.
